# Health Disparities in the Immunoprevention of Human Papillomavirus Infection and Associated Malignancies

**DOI:** 10.3389/fpubh.2015.00256

**Published:** 2015-12-17

**Authors:** Amira H. Bakir, Martin Skarzynski

**Affiliations:** ^1^George Washington University, Washington, DC, USA; ^2^National Institutes of Health, Bethesda, MD, USA

**Keywords:** health disparities, human papillomavirus, papillomavirus vaccines, cancer prevention and control, sexually transmitted diseases, herd immunity, anal cancer, cervical cancer

## Abstract

Human papillomavirus (HPV) causes roughly 1.6% of the plus 1.6 million cases of cancer that are diagnosed in the United States each year. Despite the proven safety and efficacy of available vaccines, HPV remains the most common sexually transmitted infection. Underlying the high prevalence of HPV infection is the poor adherence to the Centers for Disease Control recommendation to vaccinate all 11- to 12-year-old males and females. In fact, only about 38 and 14% of eligible females and males, respectively, receive the complete, three-dose immunization. The many factors associated with missed HPV vaccination opportunities – including race, age, family income, and patient education – contribute to widespread disparities in vaccine completion and related health outcomes. Beyond patient circumstance, however, research indicates that the rigor and consistency of recommendation by primary care providers also plays a significant role in uptake of HPV immunization. Health disparities data are of vital importance to HPV vaccination campaigns because they can provide insight into how to address current problems and allocate limited resources where they are most needed. Furthermore, even modest gains in populations with low vaccination rates may yield great benefits because HPV immunization has been shown to provide herd immunity, indirect protection for non-immunized individuals achieved by limiting the spread of an infectious agent through a population. However, the impact of current HPV vaccination campaigns is hindered by stagnant immunization rates, which remain far below target levels despite a slow overall increase. Furthermore, gains in immunization are not equally distributed across gender, age, demographic, and socioeconomic divisions within the recommended group of vaccine recipients. To achieve the greatest impact, public health campaigns should focus on improving immunization coverage where it is weakest. They should also explore more subtle but potentially significant determinants of HPV vaccine initiation and completion, such as the attitudes of parents and healthcare providers and factors that exacerbate HPV-related health outcomes, including smoking and human immunodeficiency virus-mediated immunosuppression. Optimizing the efficacy of vaccination campaigns will require a health disparities approach that both identifies and remedies the underlying causes of population differences in HPV vaccination.

## Introduction to Human Papillomavirus, HPV-Associated Cancers, and HPV Vaccines

The human papillomavirus (HPV) is the most prevalent sexually transmitted infection (STI) in the United States. Epidemiological calculations project that the majority of sexually active heterosexual males (90%) and females (85%) will be infected with HPV in their lifetimes (1). HPV infection is spread by contact with the skin and fluids of affected genital areas and through sexual activity, including vaginal, anal, and oral sex (2).

Although more than 90% of HPV infections are eventually cleared (2), HPV can evade the immune system of some individuals and cause severe, long-term health problems. The infection is associated with significantly increased risk for genital warts and a several cancers, including vaginal, vulvar, penile, anal, cervical, and oropharyngeal cancer (2). The burden of disease attributed to HPV is characterized by an annual incidence of 27,000 cases of cancer in the United States (3), including 90% of anal cancer and 70% of cervical cancer cases (4–7). Among HPV-associated malignancies, cervical cancer is of paramount concern; each year, the infection is responsible for the occurrence of 12,000 new cases of cervical cancer and the death of 4,200 American women (3).

In the United States, the incidence of cervical cancer varies by factors, such as race, ethnicity, and age. Women who are of Hispanic or Black heritage are 1.5–2 times more likely to develop cervical cancer than American women from other ethnic and racial backgrounds (8). The average morbidity for cervical cancer in Hispanic women is higher than women of other ethnicities, but this value is nearly double in African-American women (8). This fact is particularly compelling because although African-American women are less likely to develop cervical cancer than Hispanic women, they are more likely to die from the condition (8). Furthermore, recent data reveal that the rate of completion of the three-dose HPV vaccine series among African-American females actually decreased between 2013 (63.7%) and 2014 (61.6%) and remains lower than completion rates among Hispanic (72.8%), Asian (71.7%), and White (70.6%) females (9, 10). These statistics highlight the racial and ethnic disproportionalities of risk for cervical cancer among American women, and reaffirm the need to pay special attention to Hispanic and African-American girls when assessing the barriers to HPV vaccine completion.

Fortunately, protection from HPV-related STIs and cancers is available through immunization by one of three HPV vaccines that have been approved by the federal Food and Drug Administration (FDA). All three vaccines – Cervarix, Gardasil, and Gardasil 9 – are manufactured as a three-shot series administered at months 0, 1–2, and 8 (11). In general, HPV vaccination has been shown to be both safe and effective (12–18), although there are differences in the application of each vaccine. Whereas Cervarix protects against HPV types 6 and 11 and is recommended for use only in females, Gardasil additionally protects against HPV types 16 and 18 (11) and is recommended for use in both genders (19). The extensiveness of Gardasil’s protection against most HPV strains has resulted in the vaccine’s use for 99% of HPV immunization in the United States (19). The latest HPV vaccine to be approved, Gardasil 9, is even more comprehensive and protects against serotypes 31, 33, 45, 52, and 58 in addition to the strains covered by Gardasil (20, 21).

## Adverse Effects, Guidelines, and Special Considerations Related to HPV Immunization

The risk for post-vaccine injury or illness is generally a major concern for parents in their willingness to consent to the immunization of their children. Therefore, it is worthwhile to explore how the adverse effects that parents associate with HPV vaccination impact HPV vaccine uptake in adolescents. According to a Centers for Disease Control (CDC)-endorsed report by the Vaccine Adverse Event Reporting System (VAERS), a total of 25,176 adverse events were linked to HPV immunization between June 2006 and March 2014 (19). Of these reported cases, 92% were classified as “non-serious” according to the following definition: “not involving hospitalization, prolongation of an existing hospitalization, permanent disability, life-threatening illness, or death” (19). The majority of adverse effects reported for both males and females include fainting, minor reactions at injections sites (e.g., pain, swelling, and itching), headache, and nausea (19). These findings are consistent with results from studies that aggregate the adverse effects associated with HPV vaccination by gender and age distributions (19). Taken together, these data do not show significant adverse effects with the administration of HPV vaccines.

In light of the fact that 50% of adolescents become sexually active at some point during high school (ages 13–18) (22), the CDC specifically recommends the initiation of HPV vaccination between the ages of 11 and 12 to allow enough time for adolescents to complete the series and develop adequate immunity prior to HPV exposure. The advisory committee on immunization practices (ACIP) endorses the CDC’s recommendation by encouraging practitioners to incorporate the HPV vaccine into the routine immunization schedule for adolescent girls aged 11 or 12 years (23). As of 2009, ACIP has extended this recommendation to include boys in this age group (23). For adolescents who have missed the opportune window for initiating or completing HPV immunization, both the CDC and ACIP encourage catch-up immunization through age 21 and 26 for males and females, respectively (23).

However, only 38% of females and 14% of males in the recommended age group receive the complete, three-dose immunization (19). This insufficient coverage creates dangerous holes in the potential for herd immunity (24–29) against HPV infection and related diseases. Missed opportunities for HPV immunization in turn jeopardize the health of both sexually active youth and, more pressingly, vulnerable individuals who cannot be immunized (e.g., those with religious restrictions, immunodeficiency disorders, or vaccine-specific allergies). Organ transplant recipients on immunosuppressive drugs and immunosuppressed HIV patients are also of particular concern because these individuals are generally at increased risk for developing cancer (30–32), including HPV-associated malignancies (33). Furthermore, immunosuppression may preclude the development of adequate immunity to HPV even if all three recommended vaccine doses are administered.

## Healthy People 2020 Objectives, Current Progress, and Ongoing Disparities in HPV Vaccination

In the United States, the federal agency responsible for protecting the health and wellbeing of citizens is the Department of Health and Human Services (USDHHS). In assuming the task of national health promotion and disease prevention, the USDHHS has developed a public health surveillance program called Healthy People 2020 (HP2020) (34). The program’s objectives are designed as decade benchmark values for various community health indicators and are determined by the following three principles: (1) identifying the contemporary leading causes of death and illness by tracking the morbidity/mortality with specific health measures, (2) proposing evidence-based interventions for these conditions, and (3) evaluating the uptake and impact of interventions on population health (34).

Two aspects of population health that are outlined in the HP2020 mission include the promotion of reproductive health and the prevention of STIs through effective immunization (34, 35). The rampant prevalence of HPV infection coupled with inadequate HPV immunization continues to pose a major threat to the aforementioned reproductive health goals. The HP2020 target is to reach 80% completion for HPV immunization in adolescent females by 2020, a near fivefold increase from the 2008 baseline of 16.6% (19, 36). In addition to improve vaccine coverage in female adolescents, the HP2020 program seeks to address gender disparities in HPV immunization by setting vaccine coverage objectives for adolescent males (37). It is important to note that males were included in the HP2020 objectives only recently, and thus consider the current data limitations in assessing the disparities and trends specific to HPV vaccine completion in preteen and adolescent boys (37). The HP2020 objectives indicate that public health officials recognize that neglecting to immunize young males will perpetuate the spread of HPV and increase the risk to HPV-related diseases for both males and females.

Unfortunately, improvements in HPV vaccine completion rates do not align with the HP2020 goal. In fact, an increase of only 3.3% was noted for females in 2014 with little to no gains between 2011 and 2013 (9, 10, 19). Furthermore, surveillance data of HPV immunization among American adolescents indicate that several socioeconomic and demographic factors, including gender, age, race, ethnicity, regionalism, and family income level (relative to Federal Poverty Level) influence the completion of HPV immunization (38, 39). A recent report by Stokley et al. indicates that despite the fact that national HPV vaccine coverage has increased in adolescents of both genders, vaccine initiation (57 vs. 35%) and completion (38 vs. 14%) rates remain significantly higher among girls (19). In addition to gender, age plays a significant role in the completion of HPV immunization in both males and females; across all age groups (13–17), the rate of HPV immunization completion consistently increases with age (37, 40).

Researchers have also found alarming geographic differences in vaccine completion rates for females, which are lowest in the American South, particularly in Mississippi (24.6%), Arkansas (23.4%), and Tennessee (20.1%) (10). These shortcomings are not likely to be solely due to higher poverty rates in the South (4), because American adolescents whose families earn total incomes below the federal poverty level have higher HPV vaccine completion rates (9, 10). The underlying causes for these geographic differences in HPV immunization completion rates remain unknown and merit further investigation.

## Parental Attitudes toward HPV Immunization

Unfortunately, there is clear evidence that parental resistance is a barrier to HPV vaccination for children in the recommended age range of 11–12 years. One study found that parents are increasingly hesitant about vaccinating young daughters; parents are three times more likely to initiate vaccination in daughters between ages 16 and 18 than in daughters between ages 10 and 12 (41). Among adolescent boys of the same age group, vaccine initiation increases by age up to age 15 (or age 14 for 2 doses) and then fluctuates thereafter (41).

One reason for the reluctance in vaccinating preteens and younger adolescents is that parents do not want to subject their children to multiple shots during a single doctor visit (42). Despite the fact that combined vaccines have been associated with equal efficacy and similar adverse effects as single immunizations, parents are reluctant to add the HPV immunization to the meningitis and TDAP vaccines that are typically demanded by elementary/high school administrations (43). However, the bigger issue in convincing parents to vaccinate their preteen children seems to be the negative connotation associated with vaccinating young children against a virus that is transmitted through sexual contact (43). In addition to feeling, there is no need to vaccinate non-sexually active children against HPV, parents worry that discussing this risk for an STI at such a young age will influence their children’s attitudes toward abstinence. The assumption is that vaccinated adolescents may feel more protected against reproductive health risks, and thus initiate sexual activity at an earlier age (43).

The inclination to delay vaccination of young adolescents is worrisome because there is evidence that this often results in parents completely neglecting to vaccinate their children against HPV (42). Furthermore, clinical data indicate that HPV vaccination is most effective and long-lasting when all three doses are administered earlier in life. One study demonstrated that immunity granted through two doses in adolescents was temporarily non-inferior to immunity obtained by young women immunized with three doses; protection against serotypes 18 and 6 faltered at 2 and 3 years, respectively (44). While this study does not prove that two doses are sufficient for long-term protection, it provides support for immunization during early adolescence by demonstrating that immunogenicity is stronger in younger vaccine recipients. Therefore, patient age at the time of HPV vaccine initiation is likely to be an important consideration for health outcomes.

Although the HP2020 objectives focus on the completion of the full three-dose HPV vaccination series, it is important to understand disparities in the awareness and initiation of HPV vaccination as they directly influence series completion. In addition to patient age at the time of vaccine initiation, other factors that influence parental consent to HPV immunization of their children include lack of knowledge about the necessity of HPV immunization, concerns about safety and side effects, lack of recommendation by a healthcare professional, and absence of sexual activity (19, 45). Underlying these factors are two major points of dissonance in HPV vaccine education and communication: the indifference toward immunization in the parents of adolescents who are not sexually active and the belief of some parents that it is not necessary for them to vaccinate their sons.

These findings highlight the need to customize the focus of HPV vaccine campaigns to differences in the misconceptions about the reasons for vaccination of both genders. Whereas the focus for adolescent girls should clarify the safety and efficacy of HPV vaccines in the protection against HPV infection and cervical cancer, educational materials for the parents of adolescent boys should highlight the relevancy of HPV immunization of males as a means to protect both genders from HPV infection and the many different malignancies associated with this virus.

## Healthcare Provider Recommendation of HPV Vaccination

A handful of observational studies have been conducted to dissect the underlying causes of negative parental attitudes toward HPV immunization. One study by the CDC revealed that lack of vaccine recommendation by a healthcare provider is the primary reason why 13 and 23% parents do not have their daughters and sons vaccinated (19).

Clearly, the absence of a healthcare provider’s recommendation of HPV vaccination is a leading cause for missed immunization opportunities in both genders.

Another study compared parental attitudes with vaccine uptake and found that the three main reasons [“poor knowledge (about HPV and the HPV vaccine), low perceived disease susceptibility, and concerns about vaccine safety”] why parents fail to immunize their adolescents against HPV are tied to uncertainties that could be clarified by a healthcare provider (45). The same study established a positive correlation between vaccine uptake and a provider’s recommendation for immunization, but unfortunately revealed that less than half of the parents interviewed (228/487) reported discussing HPV vaccination with their physician (45). Furthermore, this study determines the frequency of vaccine-specific conversations between parents and physicians differs mostly by race, ethnicity, and maternal education; non-blacks, Hispanics, and mothers with less than a high school education reported the least number of conversations (45).

Yet, another study cross-analyzed the concerns that prevent physicians from recommending vaccines with the psychosocial assumptions that prevent parents from immunizing their adolescent daughters (42). This study reaffirms that the main reason why parents refuse to immunize their daughters against HPV is the absence of a healthcare provider’s recommendation (42). Other perceptions that present decisive barriers to vaccination in adolescent females include belief that the vaccine is optional, unnecessary, or even discouraged by physicians, not knowing enough about the vaccination, and not understanding the need to vaccinate girls as young as 11 years old (42). Among providers, the leading causes for withholding recommendations about HPV immunization are reluctance to give multiple shots in a single visit, perception of the patient’s decreased sexual activity, association of excessive emotional charge with discussing STI’s while parents are present with their children, and the prioritization of other health information that needs to be shared during the visit (42).

Another study specifically explored the efficacy of HPV education by analyzing how the content, design, and distribution channels of patient education materials influences parental attitudes toward vaccine uptake. This study finds that although half of the parents prefer written material in addition to speak with their physicians, a quarter reported difficulty in understanding written material, and the majority (88%) favored verbal communication as the ideal means of obtaining vaccine information (46). Additional analysis of online HPV information confirms that parents who primarily receive HPV information from the online sources are more likely to resist immunizing their sons. Although the majority of HPV-related websites report unbiased and reliable information about the vaccine, half of YouTube videos on HPV immunization express anti-vaccine sentiments (46). These findings suggest that physician conversation should be the main vehicle for communicating accurate vaccine information, non-verbal information should occur primarily through visual media, and simple written materials should be available to parents as optional reinforcement.

The conclusions from these studies suggest that encouraging physicians to be clear and consistent in recommending HPV vaccination can significantly increase the prevalence of favorable parental attitudes toward HPV immunization. More specifically, these recommendations should be communicated in an “ongoing discussion” that is prefaced with sufficient information about HPV and the vaccine in the two to three visits before the scheduled immunization (45). Establishing this ongoing discussion can be a valuable addition to HPV vaccine campaigns because it would give parents the time to develop informed decisions and share misconceptions about the HPV vaccine with their healthcare provider, who can in turn address these concerns before they dismiss valuable vaccination opportunities (45). Provider recommendation may be especially critical in optimizing vaccine uptake among males, who have significantly lower HPV immunization rates than their female counterparts (9, 10, 47). One study shows that 97% of parents from a given sample identified their physicians as the most-trusted authority on vaccines, and that 75% these parents consented to have their sons immunized against HPV when approached with a physician’s recommendation (46).

Given the aforementioned points about parental attitudes toward the HPV vaccine, it is evident that the role of healthcare providers as key influencers in the uptake of HPV immunization cannot be ignored in the process of redesigning the HPV education experience for parents. Practical measures for eliminating barriers and inconsistencies in the effective communication of HPV vaccine recommendation include incorporating a clear rationale for early vaccination and standardizing the content and distribution of patient education material during routine pediatric visits. If physicians feel uncomfortable prioritizing and presenting HPV immunization in front of younger adolescents, allied health personnel can be assigned to deliver this information in a private and sensitive manner. Another solution may be to present HPV vaccine information to the general patient population (without targeting individual patients) in the form of making video PSAs, posters, and pamphlets available to patients in waiting rooms.

## Impact of Community-Based Interventions on HPV Awareness and Immunization

The good news is that public health authorities are responding to the miscommunication of HPV and HPV vaccine education by creating effective campaigns that target adolescents, parents, and providers as key players in the uptake of HPV immunization. The impact of community-based health facilities is highlighted by the finding that despite an equal willingness to vaccinate male adolescents, parents who visited community health centers for vaccines were 3.5 times more likely (69 vs. 20%) to have vaccinated their sons than those who attended hospital for the same service (46). These data are further nuanced by a significant difference in the racial distribution of patients who received vaccines from hospitals (60% black) versus those who were immunized at community health centers (70% Latino) (46). These findings suggest that although community-based vaccination efforts may be a powerful approach in promoting HPV immunization, their success may depend largely on the cultural sensitivity and relevance of these patient education experiences (46).

The first of a few examples of community-based HPV immunization campaigns that will be explored in this section is “Third Time’s A Charm,” a campaign launched in 2013 by the Alabama Department of Public Health (48). The Campaign consists of a 30-s video PSA that challenges the main misconceptions about HPV immunization. The video explains that HPV vaccines protects both males and females against HPV-related cancers, emphasizes the importance of receiving all three doses, explains that the vaccine can be combined with routine immunizations, and states that the preventive service is covered by insurance (49). The program has instituted a successful birthday-card reminder system to encourage adolescents to follow-up with the second and third series shots, and reached a large audience by advertising the PSA video in movie theaters (49). However, one aspect of this campaign that should be critiqued is its reliance of feminine esthetics (e.g., pink color scheme, heart and handbag charm bracelets), which may undermine vaccine uptake in adolescent males by perpetuating the idea that HPV-protection is a focus for female health (49).

“Protect Me with 3” is a second contemporary HPV vaccine campaign. The program, pioneered by The Arizona Partnership for Immunization (TAPI), focuses on the normalization of HPV vaccination (i.e., eliminating the stigma that it is a vaccine for sexually active adolescents) by integrating it with routine vaccine recommendations (50). The Campaign recommends HPV vaccination as a third integral component of adolescent vaccination (“1” dose of TDAP, “2” doses of meningococcal, and “3” doses of HPV), and emphasizing the vaccine’s role as protection against HPV-related cancers (50). This approach can be valuable to other HPV education campaigns because it models a practical way to reeducate parents that (a) cervical cancer is not the only health risk associated with HPV and (b) males are stakeholders in the benefits of receiving the immunization. Another strength of the campaign is that it segments its target audience into teens and parents, and appropriately customizes patient education material to the assumptions and values of each group (51). By making HPV information accessible to teens, the “Protect Me with 3” program empowers adolescents to become involved in the decision-making process of HPV immunization. This tactic can be particularly instrumental in addressing the immunization disparities associated with maternal education because HPV educated adolescents are better prepared to clarify the misconceptions that ground their parents’ resistance to HPV immunization.

“You are the Key to Cancer Prevention” is a third HPV immunization campaign that places emphasis on the role of health care providers (HCPs) as key influencers on the incidence of HPV-related cancers among young Americans. The program expands HCPs’ understanding of HPV’s burden of disease by speaking to both the clinical and financial implications of missed opportunities for HPV prevention; it quantifies the morbidity and mortality of cervical cancer with compelling statistics, and attributes $7 billion to the costs of treating genital HPV among non-vaccinated American women (52). The program also clarifies the recent–most updated CDC recommendations (endorsed by the American Association of Pediatrics and ACIP) for HPV immunization, addresses the safety concerns previously associated with immunizing males, and explains the importance of correctly spacing the three doses (52). Furthermore, the program speaks to the impact of complete HPV immunization by highlighting that reaching 33% of the target objective has decreased the prevalence of cervical cancer causing HPV by 56% among female adolescents (52). Above all, this program provides HCP with a practical means of overcoming the barriers of HPV vaccine recommendation by providing conversation guides (e.g., key point checklist and Q&A dialog samples) for speaking to mothers (52).

## Conclusion

The reviewed literature has revealed that patient education is one of the most significant determinants of HPV vaccine uptake, and a leading cause for missed immunization opportunities among both males and females is the lack of vaccine recommendation by a HCP. Making sure that primary care physicians are equipped with the knowledge, cultural sensitivity, and practical guides to inform their patients about HPV immunization is essential to increase the ability of parents to make informed decisions about this vaccine and their willingness to accept it for their children. Realizing that primary care physicians are often tasked with covering an overwhelming amount of information during physical exams, it may be optimal to incorporate patient-led education initiatives in the form of virtual media (e.g., PSAs) and take-home material (e.g., pamphlets, links to websites, and social media pages).

Health disparities in the HPV vaccination coverage are a serious public health concern but can be looked as an opportunity to make significant progress in the United States population as a whole. HPV vaccination should be carefully approached from a health disparities perspective because some disparities are not detectable at the level of vaccine initiation and are only evident when data on the completion of immunization are considered (53). The latest available suggest that income disparities are particularly acute, with lower immunization rates below the federal poverty level at every dose (1–3) in both males and females (10). These data are particularly concerning when considering disparities that smoking rates are much higher below the poverty level (54), as smoking is known to contribute the risk and exacerbation of malignancies resulting from HPV infection (55–59).

However, the demonstrated herd immunity effect in HPV vaccination (24–29) suggests that vaccine coverage need not be universal for effective population protection. This suggests diminishing returns as HPV vaccination coverage nears completion, but effective herd immunity may require high levels of immunization depending on the characteristics of the vaccine and infectious agent (60, 61). HPV vaccination rates in the United States are far from perfect and available data suggest that improvements in HPV immunization can yield tremendous public health benefits. An example of a country that has made impressive gains in HPV vaccination is Rwanda, which has achieved complete immunization coverage since the introduction of the HPV vaccine in 2011 (62, 63). The success of these countries demonstrates that the goals set by the Healthy People 2020 initiative are attainable and should be enthusiastically pursued.

Parallels can be drawn between modern HPV vaccination efforts and previous work that led to the eradication of polio in the United States in 1979. Similar to HPV vaccine development, the poliovirus vaccine was also improved in a stepwise manner with the increasing valency of new iterations (64, 65). Another similarity is that there is strong evidence to support the safety and efficacy of both the HPV (12, 13, 20, 28) and poliovirus vaccines (66–75), even though the extent to which herd immunity is responsible for their efficacy can still be debated. These resemblances, as well as the data and programs reviewed herein, offer hope that HPV vaccination programs achieve the same degree of success as polio vaccination. Continued emphasis on the successful implementation of the immunization strategies may 1 day lead to the global eradication of both HPV and the poliovirus.

## Author Contributions

All authors contributed to the writing of this literature review. All authors reviewed and approved the final version.

## Conflict of Interest Statement

The authors declare that the research was conducted in the absence of any commercial or financial relationships that could be construed as a potential conflict of interest.
